# Reduced Sleep Duration and Sleep Efficiency Were Independently Associated With Frequent Nightmares in Chinese Frontline Medical Workers During the Coronavirus Disease 2019 Outbreak

**DOI:** 10.3389/fnins.2020.631025

**Published:** 2021-01-20

**Authors:** Yi-Qi Lin, Ze-Xin Lin, Yong-Xi Wu, Lin Wang, Zhao-Nan Zeng, Qiu-Yang Chen, Ling Wang, Xiao-Liang Xie, Shi-Chao Wei

**Affiliations:** ^1^Department of Sleep Center, Fujian Provincial Hospital, Fujian Medical University, Fuzhou, China; ^2^Xinjiang Medical University Second Clinical College, Ürümqi, China; ^3^Administrative Office, Fujian Provincial Hospital, Fujian Medical University, Fuzhou, China; ^4^Center for Experimental Research in Clinical Medicine, Fujian Provincial Hospital, Fujian Medical University, Fuzhou, China; ^5^The First Operating Room, Fujian Provincial Hospital, Fujian Medical University, Fuzhou, China; ^6^Department of Pharmacy, Fujian Provincial Hospital, Fujian Medical University, Fuzhou, China

**Keywords:** sleep duration, sleep efficiency, frequent nightmares, frontline medical workers, COVID-19

## Abstract

**Objectives:**

Nightmares were related to emotion and behavioral problems and also emerged as one of the core features of post-traumatic stress disorder (PTSD). Our study aimed to investigate the associations of frequent nightmares with sleep duration and sleep efficiency among frontline medical workers in Wuhan during the coronavirus disease 2019 (COVID-19) outbreak.

**Methods:**

A total of 528 health-care workers from the province of Fujian providing medical aid in Wuhan completed the online questionnaires. There were 114 doctors and 414 nurses. The age, sex, marital status, and work situation were recorded. A battery of scales including the Pittsburgh Sleep Quality Index (PSQI) and the 12-item General Health Questionnaire (GHQ-12) were used to evaluate subjects’ sleep and general mental health. Frequent nightmares were defined as the response of at least once a week in the item of “nightmare” of PSQI.

**Results:**

Frequent nightmares were found in 27.3% of subjects. The frequent nightmare group had a higher score of PSQI-sleep duration and PSQI-habitual sleep efficiency (frequent nightmares vs. non-frequent nightmares: PSQI-sleep duration, 1.08 ± 0.97 vs. 0.74 ± 0.85, *P* < 0.001; PSQI-habitual sleep efficiency, 1.08 ± 1.10 vs. 0.62 ± 0.88, *P* < 0.001). Reduced sleep duration and reduced sleep efficiency were independently associated with frequent nightmares after adjustment for age, sex, poor mental health, and regular sleeping medication use (reduced sleep duration: OR = 1.96, 95% CI = 1.07–3.58, *P* = 0.029; reduced sleep efficiency: OR = 2.17, 95% CI = 1.09–4.32, *P* = 0.027). Subjects with both reduced sleep duration and sleep efficiency were also associated with frequent nightmares (OR = 2.70, 95% CI = 1.57–4.65, *P* < 0.001).

**Conclusion:**

The present study found that sleep duration and sleep efficiency were both independently associated with frequent nightmares among frontline medical workers in Wuhan during the COVID-19 pandemic. We should pay attention to nightmares and even the ensuing PTSD symptoms among subjects with reduced sleep duration or sleep efficiency facing potential traumatic exposure.

## Introduction

As coronavirus disease 2019 (COVID-19) broke out, medical workers were under great stress, and worldwide attention was focused on their sleep characteristics. Nightmares were one of parasomnias, which present as vivid dreams containing negative emotions during rapid eye movement (REM) with usually subsequent awakenings (Sateia, 2014). During the COVID-19 pandemic, nightmares were found in 38–59% (Giardino et al., 2020; Herrero San Martin et al., 2020; Tu et al., 2020) of health-care workers at the frontline, higher than those in non-health-care workers (Herrero San Martin et al., 2020). Nightmares were related to depression symptoms, anxiety disorder, psychiatric diagnosis, and neuroticism personality trait in the general public (Nakajima et al., 2014; Sandman et al., 2015). More importantly, nightmares emerged as one of the core features of post-traumatic stress disorder (PTSD) (Spoormaker and Montgomery, 2008). For example, nightmares and other disruptive nocturnal behaviors were found in PTSD patients exposed to the L’Aquila earthquake including not only L’Aquila citizens but also those living in proximity to the epicenter (Tempesta et al., 2013). Nightmares were also found to be associated with mental and behavioral problems including depression and self-destructive behavior (Sateia, 2014) and were reported to predict PTSD symptoms dependent on mood and anxiety symptoms (van Liempt et al., 2013). Nightmares in PTSD are often resistant to first-line treatment, which is effective for some other subjective sleep complaints (Germain, 2013). Though almost half of PTSD cases resolve within 3 months, nightmares could persist in one’s entire life (Sateia, 2014). Dealing with COVID-19 is potential trauma exposure for health-care workers, who were found to be at higher risk of PTSD after the outbreak of severe acute respiratory syndrome (SARS) in 2003 and Middle East respiratory syndrome coronavirus (MERS-Cov) in 2012 (Carmassi et al., 2020). Therefore, nightmares in frontline medical workers were worthy of attention during the COVID-19 pandemic.

Insomnia was found to be significantly associated with nightmares in the general adult population (Sandman et al., 2015). Subjects with insomnia symptoms always have an impression of insufficient or inefficient sleep, which could be measured by sleep duration and sleep efficiency. As sleep efficiency was calculated as the ratio of sleep duration to total time in bed, these two parameters were in relation but not always consistent. A “U-shape” relationship between sleep duration and nightmares was previously found, which meant that frequent nightmares were associated with both decreased and increased sleep duration in adolescents (Munezawa et al., 2011) and also in adults (Sandman et al., 2015). The association between sleep efficiency and nightmares was less studied and remained controversial. Nightmares may promote more arousal, and in turn, subjects with light sleep tend to have a higher possibility of nightmares (van Wyk et al., 2019). In the elderly, awakenings from bad dreams were found to be correlated with decreased sleep efficiency (Desjardins et al., 2019). However, decreased sleep efficiency was not reported to be related to nightmares in military veterans with PTSD (Miller et al., 2018).

To our best knowledge, the associations of nightmares with sleep duration or sleep efficiency during the COVID-19 pandemic have not been studied. We hypothesized that decreased sleep efficiency and sleep duration were both independently associated with frequent nightmares. Our study aimed to investigate the associations of frequent nightmares with sleep duration and sleep efficiency among frontline medical workers in Wuhan dealing with the COVID-19 pandemic.

## Materials and Methods

### Study Design and Subjects

This cross-sectional study was conducted from March 19 to April 15, 2020, through a program named Questionnaire Star, which was widely used in China. The study was approved by the Ethics Committee of Fujian Provincial Hospital. We invited frontline health-care workers from the province of Fujian dealing with COVID-19 patients in hospitals in Wuhan, China, to participate in our investigation through the link or Quick Response (QR) code of Questionnaire Star with a non-probability convenience-sampling design. The inclusion criteria were as follows: (1) doctors or nurses coming from the province of Fujian to provide medical aid in Wuhan to fight COVID-19; and (2) medical workers dealing with COVID-19 patients in hospitals in Wuhan. The questionnaire could only be submitted when all the questions were answered. The ethics committee waived the requirement of written informed consent for participation. All the data were saved in the Questionnaire Star server and could be viewed or downloaded by the developer. Finally, 528 health-care workers including 114 doctors and 414 nurses completed the questionnaires.

### Measures

#### Demographic Characteristics

The age, sex, and marital status (single, married, and divorced or others) were recorded. Information about working status included the following: occupation type (doctors or nurses); frequency of shift work, which was defined as working from 8 p.m. to 8 a.m.; the number of working hours per day; and the number of working days per week.

#### Measure of Sleep States

The Pittsburgh Sleep Quality Index (PSQI) scale was used to assess sleep quality and investigate sleep disturbance, which consisted of seven components (Buysse et al., 1989). Nightmare was assessed by item of “nightmare” in component 5 of PSQI. The question was “How often did you have nightmare in the previous month?” The answers to this question included “not during the past month,” “less than once a week,” “once or twice a week,” and “three or more times a week.” No definition of a nightmare was provided. Subjects who answered “once or twice a week” and “three or more times a week” were considered to have frequent nightmares, consistent with previous reports (Li et al., 2010; Nakajima et al., 2014). Then subjects were divided into groups of “frequent nightmares” and “non-frequent nightmares” according to the presence of frequent nightmares.

Sleep duration and sleep efficiency were investigated by component 3 and component 4 of PSQI, respectively. The question of sleep duration was “How long did you sleep every night?” Sleep duration of less than 7 h was considered to be reduced sleep duration (Bandyopadhyay and Sigua, 2019). Subjects with sleep duration of no less than 7 h were treated as normal, since only 3.9% of them slept more than 9 h. Habitual sleep efficiency was calculated as the ratio of sleep duration to total time in bed and categorized as “more than 85%,” “75–84%,” “65–74%,” and “less than 65%.” Sleep efficiency of less than 85% was thought to be reduced sleep efficiency. Habitual use of sleeping medication was defined as at least once a week in component 6 of PSQI.

Besides, the Epworth Sleepiness Scale (ESS) was used to evaluate daytime sleepiness symptoms (Johns, 1991). The chronotype was assessed by the reduced version of Morningness–Eveningness Questionnaire (rMEQ), which consisted of five items (Adan and Almirall, 1991). A score lower than 12 was defined as evening type, and higher than 17 was considered as morning type. The intermediate type was considered among subjects with a score between 12 and 17, consistent with previous reports in China (Carciofo et al., 2012; Li et al., 2018).

#### Measure of Emotional Problem

Subjects were required to choose the number from 0 to 10 to assess the degree of “worry about being infected with COVID-19,” “worry about family’s infection of COVID-19,” “confidence in being cured if infected,” and “sense of competence in frontline medical work.” In addition, the 12-item General Health Questionnaire (GHQ-12) was used to evaluate general mental health (Goldberg, 1972), which was validated in China (Lee et al., 2006; Li et al., 2009); and subjects who scored higher than 2 were thought to be in poor mental health, consistent with previous reports (Goldberg and Pa, 1988; Lee et al., 2007).

### Statistical Analysis

Continuous and categorical data were presented as mean with standard deviation (SD) and percentage, respectively. Comparisons of demographic characteristics, and sleep and emotional assessment between subjects with and without frequent nightmares were conducted by chi square analysis for categorical variables and Wilcoxon rank sum for continuous variables after testing normality. Logistic regression analysis was used to investigate the association of reduced sleep duration and sleep efficiency with frequent nightmares. Odds ratios (ORs) and 95% confidence intervals (CIs) were calculated. In model 1, age and sex were adjusted. Then model 2 was further adjusted for poor mental health and habitual sleeping medication use on the basis of model 1. SPSS 22.0 (IBM, Armonk, NY, United States) was used to perform all statistical analyses. Overall statistical significance level was set at a *P*-value of <0.05 with two sides.

## Results

### Demographic Characteristics of Subjects With and Without Frequent Nightmares

Frequent nightmares were found in 27.3% of subjects. The age was significantly lower in the frequent nightmare group (frequent nightmares vs. non-frequent nightmares: 32.29 ± 5.77 vs. 34.13 ± 6.52; *P* = 0.004) (Table 1). No difference was found between these two groups in marital status (*P* = 0.061), occupation type (*P* = 0.227), frequency of shift work (*P* = 0.185), the number of working hours per day (*P* = 0.121), or working days per week (*P* = 0.145).

**TABLE 1:** Demographic characteristics of subjects with and without frequent nightmares.

	**Frequent nightmares**	**Non-frequent nightmares**	***P***
	***n* = 144**	***n* = 384**	
Age, mean (SD)	32.29 (5.77)	34.13 (6.52)	0.004
Sex (male, n, %)	30 (20.8%)	103 (26.8%)	0.158
**Marital status**			
Single, n, %	53 (36.8%)	118 (30.7%)	0.061
Married, n, %	89 (61.8%)	244 (63.5%)	
Divorced or others, n, %	2 (1.04%)	22 (5.7%)	
Occupation type (doctors, n, %)	26 (18.1%)	88 (22.9%)	0.227
**Frequency of shift work**			
≤3/week, n, %	112 (77.8%)	318 (82.8%)	0.185
>3/week, n, %	32 (22.2%)	66 (17.2%)	
Working hours per day, mean (SD)	8.51 (2.15)	8.33 (4.91)	0.121
Working days per week, mean (SD)	4.93 (1.20)	5.09 (1.29)	0.145

### Sleep and Emotional Assessment of Subjects With and Without Frequent Nightmares

Compared with the score of the non-frequent nightmare group, the score of the frequent nightmare group was significantly higher in worry about being infected and family’s infection of COVID-19 (frequent nightmares vs. non-frequent nightmares: worry about being infected, 5.79 ± 2.47 vs. 4.71 ± 2.81, *P* < 0.001; worry about family’s infection, 6.07 ± 2.94 vs. 5.08 ± 3.14, *P* = 0.001). The score of confidence in being cured if infected and sense of competence was significantly lower in the frequent nightmare group (frequent nightmares vs. non-frequent nightmares: confidence, 8.02 ± 1.89 vs. 8.39 ± 1.74, *P* = 0.031; competence, 7.76 ± 1.83 vs. 8.31 ± 1.59, *P* = 0.002). The percentage of poor mental health in the frequent nightmare group was significantly higher (frequent nightmares vs. non-frequent nightmares: 20.8% vs. 10.2%, *P* = 0.001). The frequent nightmare group had a higher score in GHQ-12 (frequent nightmares vs. non-frequent nightmares: 1.51 ± 1.55 vs. 0.87 ± 1.22; *P* < 0.001) (Table 2).

**TABLE 2:** Sleep and emotional assessment in participants with and without frequent nightmares.

	**Frequent nightmares**	**Non-frequent nightmares**	***P***
	***n* = 144**	***n* = 384**	
**Emotional assessment**			
Worry about being infected of COVID, mean (SD)	5.79 (2.47)	4.71 (2.81)	< 0.001
Worry about family members’ infection of COVID, mean (SD)	6.07 (2.94)	5.08 (3.14)	0.001
Confidence in being cured if infected, mean (SD)	8.02 (1.89)	8.39 (1.74)	0.031
Sense of competence, mean (SD)	7.76 (1.83)	8.31 (1.59)	0.002
GHQ-12, mean (SD)	1.51 (1.55)	0.87 (1.22)	< 0.001
Poor mental health, n, %	30 (20.8%)	39 (10.2%)	0.001
**Sleep assessment**			
Sleep duration every night, mean (SD)	6.08 (1.38)	6.63 (1.19)	< 0.001
ESS, mean (SD)	6.84 (5.09)	5.15 (3.86)	0.001
Chronotype			
Morning type, n, %	30 (20.8%)	60 (15.6%)	0.112
intermediate type, n, %	94 (65.3%)	244 (63.5%)	
Evening type, n, %	20 (13.9%)	80 (20.8%)	
PSQI, mean (SD)	9.33 (3.50)	6.00 (3.48)	< 0.001
PSQI-subjective sleep quality, mean (SD)	1.71 (0.68)	1.22 (0.68)	< 0.001
PSQI-sleep latency, mean (SD)	2.15 (0.79)	1.60 (0.95)	< 0.001
PSQI-sleep duration, mean (SD)	1.08 (0.97)	0.74 (0.85)	< 0.001
PSQI-habitual sleep efficiency, mean (SD)	1.08 (1.10)	0.62 (0.88)	< 0.001
PSQI-sleep disturbance, mean (SD)	1.73 (0.57)	1.03 (0.51)	< 0.001
PSQI-use of sleep medication, mean (SD)	0.79 (1.13)	0.33 (0.77)	< 0.001
PSQI-daytime dysfunction, mean (SD)	0.80 (1.14)	0.46 (0.81)	0.002

*COVID, coronavirus disease; GHQ-12, 12-item General Health Questionnaire; ESS, Epworth Sleepiness Scale; PSQI, Pittsburgh Sleep Quality Index.*

Sleep duration every night was significantly decreased in the frequent nightmare group (frequent nightmares vs. non-frequent nightmares: 6.08 ± 1.38 vs. 6.63 ± 1.19, *P* < 0.001). The ESS score was found to be higher in the frequent nightmare group (frequent nightmares vs. non-frequent nightmares: 6.84 ± 5.09 vs. 5.15 ± 3.86, *P* = 0.001). No difference was found between two groups in chronotype (*P* = 0.112). The frequent nightmare group had a higher score in PSQI, PSQI-sleep duration, and PSQI-habitual sleep efficiency (frequent nightmares vs. non-frequent nightmares: PSQI, 9.33 ± 3.50 vs. 6.00 ± 3.48, *P* < 0.001; PSQI-sleep duration, 1.08 ± 0.97 vs. 0.74 ± 0.85, *P* < 0.001; PSQI-habitual sleep efficiency, 1.08 ± 1.10 vs. 0.62 ± 0.88, *P* < 0.001). The score of the other PSQI components was also higher in the frequent nightmare group including subjective sleep quality, sleep latency, sleep disturbance, use of sleeping medication, and daytime dysfunction (Table 2).

### Sleep Duration and Sleep Efficiency in Relation to Frequent Nightmares

**FIGURE 1:**
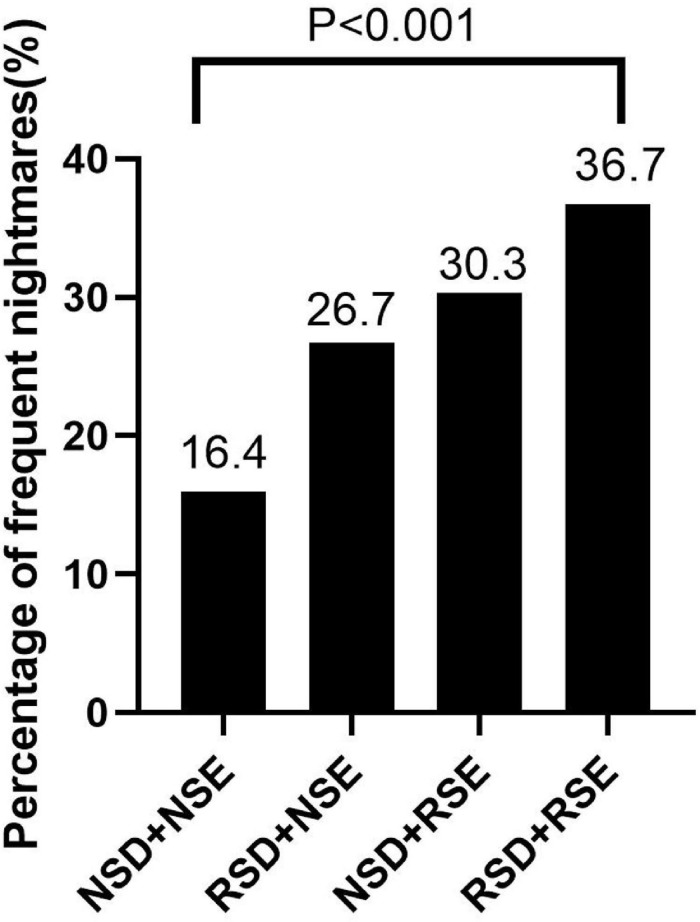
Presence of reduced sleep duration or sleep efficiency in relation to frequent nightmares (*P* < 0.001, linear-by-linear association). NSD, normal sleep duration; NSE, normal sleep efficiency; RSD, reduced sleep duration; RSE, reduced sleep efficiency.

Frequent nightmares were found in 16.4% of subjects with normal sleep duration and sleep efficiency and 26.7% of only reduced sleep duration subjects, while the percentage increased to 30.3% in only reduced sleep efficiency group and 36.7% in both reduced sleep duration and sleep efficiency group (*P* < 0.001, linear-by-linear association) (Figure 1). No difference was found in the percentage of poor mental health between four groups (*P* = 0.233). As demonstrated in Table 3, the presence of reduced sleep duration only and the presence of reduced sleep efficiency only were two independent factors in relation to frequent nightmares in model 1 after adjustment for age and sex (reduced sleep duration only: OR = 2.10, 95% CI = 1.16–3.79, *P* = 0.014; reduced sleep efficiency only: OR = 2.11, 95% CI = 1.07–4.14, *P* = 0.031) and also in model 2 with adjustment for age, sex, poor mental health, and regular sleeping medication use (reduced sleep duration only: OR = 1.96, 95% CI = 1.07–3.58, *P* = 0.029; reduced sleep efficiency only: OR = 2.17, 95% CI = 1.09–4.32, *P* = 0.027). Subjects with both reduced sleep duration and sleep efficiency were associated with frequent nightmares in model 1 (OR = 3.18, 95% CI = 1.88–5.37, *P* < 0.001) and also in model 2 (OR = 2.70, 95% CI = 1.57–4.65, *P* < 0.001).

**TABLE 3:** Sleep duration and sleep efficiency in relation to frequent nightmares.

**Variable**	**Model 1^a^**		**Model 2^b^**	
	**OR (95% CI)**	**P**	**OR (95% CI)**	**P**
Normal sleep duration and sleep efficiency				
Only reduced sleep duration	2.10 (1.16–3.79)	0.014	1.96 (1.07–3.58)	0.029
Only reduced sleep efficiency	2.11 (1.07–4.14)	0.031	2.17 (1.09–4.32)	0.027
Reduced sleep duration + reduced efficiency	3.18 (1.88–5.37)	< 0.001	2.70 (1.57–4.65)	< 0.001

*^*a*^Adjusted for age and sex. ^*b*^Adjusted for age, sex, poor mental health, and habitual sleeping medication use.*

## Discussion

To our knowledge, our study found for the first time that reduced sleep duration and reduced sleep efficiency were independently associated with frequent nightmares among frontline medical workers dealing with COVID-19 in Wuhan. Also, the presence of both reduced sleep duration and sleep efficiency together was in relation to frequent nightmares.

Frequent nightmares were found in 2–6% of the general population (Li et al., 2010; Schredl, 2010; Sandman et al., 2015) and 27.3% of medical workers dealing with COVID-19 patients in the present study. Compared with our findings, previous reports found a higher prevalence (38–59%) of nightmares in frontline health-care workers during the COVID-19 pandemic (Giardino et al., 2020; Herrero San Martin et al., 2020; Tu et al., 2020). The discrepancy may mainly come from differences in frequency required for diagnosing a nightmare, which was not described or was defined as the response of “yes” of “presenting nightmare symptoms” in some studies. Besides, a “nightmare” was not defined in most studies, which was still controversial, including arguments on the requirement of fear and awakenings from dreams for the diagnosis (Zadra and Donderi, 2000; Blagrove and Haywood, 2006). Therefore, participants may have a different understanding due to differences in their cultural background and educational degree.

Sleep duration and sleep efficiency were two associated sleep parameters that assessed the quantity and quality of sleep, respectively. Sleep efficiency represented difficulty in falling asleep and staying asleep (Spielman et al., 1987), while sleep duration was time falling asleep in total. Most previous studies chose one of the two parameters to investigate its relation with a nightmare. As sleep efficiency was calculated as sleep duration divided by time in bed, these two sleep parameters were highly associated but not always consistent. Our findings demonstrated that reduced sleep duration and sleep efficiency were two independent factors in relation with frequent nightmares among frontline medical workers. Sleep efficiency represents “sleep continuity” and was often the primary outcome measure in insomnia research. It remained controversial over its association with nightmares. An actigraphy study found no association between sleep efficiency and nightmares in military veterans with PTSD (Miller et al., 2018). However, a meta-analysis of polysomnographic studies found that sleep efficiency was reduced among patients with PTSD (Zhang et al., 2019) and that REM interruption was also reported to correlate with nightmare complaints (Habukawa et al., 2018). Consistent with polysomnographic studies, our study also found the association between reduced sleep efficiency and frequent nightmares. However, our present cross-sectional study had a difficulty in determining their causal relationship, and there may exist a vicious cycle. Frequent nightmares may partly result in decreased sleep efficiency through more nocturnal awakenings. For example, there were more awakenings reported on nights with nightmares than nights without in PTSD patients (Gehrman et al., 2015). In addition, fear of nightmares was found to be associated with insomnia symptoms (Hall Brown and Mellman, 2014), which may aggravate decreased sleep efficiency in subjects with frequent nightmares. In turn, subjects with light or fragmented sleep may be more likely to recall their dreams, evidenced by more non-rapid eye movement (NREM) awakenings (van Wyk et al., 2019) and specific electroencephalograph (EEG) findings of lower delta in parietal or other areas during NREM (Scarpelli et al., 2017, 2020) reported among frequent dream recallers compared with non-frequent dream recallers. Therefore, reduced sleep efficiency may also contribute to recalls of dreams including nightmares. Their causal relationship should be further investigated.

Reduced sleep duration was also found to be associated with nightmares in the present study. In the general population, increasing evidence has shown the association of longer and shorter sleep duration with nightmares among adolescents and adults (Munezawa et al., 2011; Sandman et al., 2015; Rek et al., 2017; Lin et al., 2020). Increased and decreased sleep durations were also both reported to be associated with PTSD symptoms (Usami et al., 2013; Gehrman et al., 2015; McCall et al., 2019). Among PTSD patients, sleep duration on nights with nightmares were significantly decreased than that on nights without nightmares (Gehrman et al., 2015). Besides, there was an association between shorter sleep duration and greater functional interference due to nightmares in PTSD patients (Gehrman et al., 2015). Consistently, reduced sleep duration was found to be related to frequent nightmares in our study, independently of reduced sleep efficiency. REM rebound may be one of underlying mechanisms. Increased REM duration was found in extended sleep and also in recovery nights after sleep deprivation in adults (Reynolds et al., 1986; Rupp et al., 2009; Arnal et al., 2015). Nightmare, as one of parasomnias in REM stage, was more likely to occur on recovery nights such as off days with REM rebound (Reynolds et al., 1986). In addition, nightmares and some other PTSD symptoms were thought to be partly a result of autonomic nervous dysfunction, as modulating autonomic nervous system could treat PTSD or increase the risk of developing PTSD (Williamson et al., 2013). Interestingly, REM deprivation was found to unmask altered heart rate variability in subjects with frequent nightmares (Nielsen et al., 2010). Therefore, another hypothesis was that sleep deprivation may precipitate the occurrence of a nightmare among subjects with potential autonomic dysregulation when faced with traumatic exposure. Increased sleep duration was not investigated in our study because of its low prevalence in our sampling, which was proposed to associate with increased dream recalls during the COVID-19 pandemic through sleep fragmentation (Bottary et al., 2020).

There were several limitations in our study. Firstly, the convenience sampling may lack generalizability, although it was easy and readily available to be used among frontline medical workers during the COVID-19 outbreak. Secondly, sleep duration and sleep efficiency data were obtained from scales but not objective recordings by polysomnography or actigraphy. However, it was not practical to apply objective measurements among frontline doctors and nurses during the COVID-19 pandemic. Thirdly, stress, depression, and anxiety may be important confounding factors, which should be evaluated with more comprehensive measures. To address time-consuming concern, we only used GHQ-12 scale to assess general mental health of our medical workers, which was also adjusted in finding the association of nightmares with sleep duration and efficiency. Lastly, the present cross-sectional study could not determine the causal relationship; however, the association was highlighted between frequent nightmares and reduced sleep duration as well as sleep efficiency, which needed further prospective studies.

In summary, our study found that reduced sleep duration and sleep efficiency were both independently associated with frequent nightmares among frontline medical workers in Wuhan during the COVID-19 pandemic. Therefore, we should pay attention to the occurrence of a nightmare and even the ensuing PTSD symptoms among subjects having reduced sleep duration or sleep efficiency with potential traumatic exposure. More studies will be needed to further investigate their causal relationship and to provide more precise intervention and support for frontline medical workers.

## Data Availability Statement

The raw data supporting the conclusions of this article will be made available by the authors, without undue reservation.

## Ethics Statement

The studies involving human participants were reviewed and approved by the Ethics Committee of Fujian Provincial Hospital. The ethics committee waived the requirement of written informed consent for participation.

## Author Contributions

Y-QL contributed to the study design, data collection, data analysis, and writing. Z-XL and Y-XW contributed to the study design, data collection, and data analysis. LW, Z-NZ, Q-YC, LW, and X-LX contributed to the study design and data collection. S-CW supervised, conceptualized, and reviewed the manuscript. All authors contributed to the article and approved the submitted version.

## Conflict of Interest

The authors declare that the research was conducted in the absence of any commercial or financial relationships that could be construed as a potential conflict of interest.

## References

[B1] Adan A. Almirall H. (1991). Horne & Östberg morningness-eveningness questionnaire: a reduced scale. *Pers. Indiv. Differ.* 12 241–253.

[B2] Arnal P. J. Sauvet F. Leger D. van Beers P. Bayon V. Bougard C. (2015). Benefits of sleep extension on sustained attention and sleep pressure before and during total sleep deprivation and recovery. *Sleep* 38 1935–1943. 10.5665/sleep.5244 26194565 PMC4667385

[B3] Bandyopadhyay A. Sigua N. L. (2019). What is sleep deprivation? *Am. J. Respir. Crit. Care Med.* 199 11–12. 10.1164/rccm.1996P11 30874458

[B4] Blagrove M. Haywood S. (2006). Evaluating the awakening criterion in the definition of nightmares: how certain are people in judging whether a nightmare woke them up? *J. Sleep Res.* 15 117–124. 10.1111/j.1365-2869.2006.00507.x 16704565

[B5] Bottary R. Simonelli G. Cunningham T. J. Kensinger E. A. Mantua J. (2020). Sleep extension: an explanation for increased pandemic dream recall? *Sleep* 43:zsaa131. 10.1093/sleep/zsaa131 32886777

[B6] Buysse D. J. Reynolds C. F. III Monk T. H. Berman S. R. Kupfer D. J. (1989). The Pittsburgh Sleep Quality Index: a new instrument for psychiatric practice and research. *Psychiatry Res.* 28 193–213. 10.1016/0165-1781(89)90047-4 2748771

[B7] Carciofo R. Du F. Song N. Qi Y. Zhang K. J. S. Rhythms B. (2012). Age−related chronotype differences in Chinese, and reliability assessment of a reduced version of the Chinese Morningness–Eveningness Questionnaire. *Sleep Biol. Rhythms* 10 310–318. 10.1111/j.1479-8425.2012.00577.x

[B8] Carmassi C. Foghi C. Dell’Oste V. Cordone A. Bertelloni C. A. Bui E. (2020). PTSD symptoms in healthcare workers facing the three coronavirus outbreaks: what can we expect after the COVID-19 pandemic. *Psychiatry Res.* 292:113312. 10.1016/j.psychres.2020.113312 32717711 PMC7370915

[B9] Desjardins S. Lapierre S. Hudon C. Desgagné A. (2019). Factors involved in sleep efficiency: a population-based study of community-dwelling elderly persons. *Sleep* 42:zsz038. 10.1093/sleep/zsz038 30768200 PMC6519908

[B10] Gehrman P. R. Harb G. C. Cook J. M. Barilla H. Ross R. J. (2015). Sleep diaries of Vietnam War veterans with chronic PTSD: the relationships among insomnia symptoms, psychosocial stress, and nightmares. *Behav. Sleep Med.* 13 255–264. 10.1080/15402002.2014.880344 24617942

[B11] Germain A. (2013). Sleep disturbances as the hallmark of PTSD: where are we now? *Am. J. Psychiatry* 170 372–382. 10.1176/appi.ajp.2012.12040432 23223954 PMC4197954

[B12] Giardino D. L. Huck-Iriart C. Riddick M. Garay A. (2020). The endless quarantine: the impact of the COVID-19 outbreak on healthcare workers after three months of mandatory social isolation in Argentina. *Sleep Med.* 76 16–25. 10.1016/j.sleep.2020.09.022 33059247 PMC7518855

[B13] Goldberg D. P. (1972). *The Detection of Psychiatric Illness By Questionnaire: A Technique for the Identification and Assessment of Non-Psychotic Psychiatric Illness.* Oxford: U Press.

[B14] Goldberg D. P. Pa W. (1988). *A User’s Guide to the General Health Questionnaire.* London: NFER.

[B15] Habukawa M. Uchimura N. Maeda M. Ogi K. Hiejima H. Kakuma T. (2018). Differences in rapid eye movement (REM) sleep abnormalities between posttraumatic stress disorder (PTSD) and major depressive disorder patients: REM interruption correlated with nightmare complaints in PTSD. *Sleep Med.* 43 34–39. 10.1016/j.sleep.2017.10.012 29482809

[B16] Hall Brown T. Mellman T. A. (2014). The influence of PTSD, sleep fears, and neighborhood stress on insomnia and short sleep duration in urban, young adult, African Americans. *Behav. Sleep Med.* 12 198–206. 10.1080/15402002.2013.784704 23767868 PMC3966964

[B17] Herrero San Martin A. Parra Serrano J. Diaz Cambriles T. Arias Arias E. M. Muñoz Méndez J. Del Yerro Álvarez M. J. (2020). Sleep characteristics in health workers exposed to the COVID-19 pandemic. *Sleep Med.* 75 388–394. 10.1016/j.sleep.2020.08.013 32950884 PMC7429626

[B18] Johns M. W. (1991). A new method for measuring daytime sleepiness: the Epworth sleepiness scale. *Sleep* 14 540–545. 10.1093/sleep/14.6.540 1798888

[B19] Lee A. M. Wong J. G. McAlonan G. M. Cheung V. Cheung C. Sham P. C. (2007). Stress and psychological distress among SARS survivors 1 year after the outbreak. *Can. J. Psychiatry* 52 233–240. 10.1177/070674370705200405 17500304

[B20] Lee D. T. Yip W. C. Chen Y. Meng Q. Kleinman A. (2006). Ethno-psychometric evaluation of the General Health Questionnaire in rural China. *Psychol. Med.* 36 249–255. 10.1017/s0033291705006434 16303061

[B21] Li S. X. Chan N. Y. Man Yu M. W. Lam S. P. Zhang J. Yan Chan J. W. (2018). Eveningness chronotype, insomnia symptoms, and emotional and behavioural problems in adolescents. *Sleep Med.* 47 93–99. 10.1016/j.sleep.2018.03.025 29778920

[B22] Li S. X. Zhang B. Li A. M. Wing Y. K. (2010). Prevalence and correlates of frequent nightmares: a community-based 2-phase study. *Sleep* 33 774–780. 10.1093/sleep/33.6.774 20550018 PMC2880244

[B23] Li W. H. Chung J. O. Chui M. M. Chan P. S. (2009). Factorial structure of the Chinese version of the 12-item General Health Questionnaire in adolescents. *J. Clin. Nurs.* 18 3253–3261. 10.1111/j.1365-2702.2009.02905.x 19732241

[B24] Lin Y. Q. Zhuang W. J. Zheng F. H. Zeng Z. N. Wu Y. X. Wu S. Y. (2020). Weekday and weekend sleep deprivation are associated with recurrent nightmare in adolescents: a cross-sectional study. *Sleep Med.* 76 36–42. 10.1016/j.sleep.2020.09.016 33075612

[B25] McCall C. A. Turkheimer E. Tsang S. Avery A. Duncan G. E. Watson N. F. (2019). Sleep duration and post-traumatic stress disorder symptoms: a twin study. *Sleep* 42:zsz179. 10.1093/sleep/zsz179 31408518

[B26] Miller K. E. Jamison A. L. Gala S. Woodward S. H. (2018). Two independent predictors of nightmares in posttraumatic stress disorder. *J. Clin. Sleep Med.* 14 1921–1927. 10.5664/jcsm.7494 30373691 PMC6223551

[B27] Munezawa T. Kaneita Y. Osaki Y. Kanda H. Ohtsu T. Suzuki H. (2011). Nightmare and sleep paralysis among Japanese adolescents: a nationwide representative survey. *Sleep Med.* 12 56–64. 10.1016/j.sleep.2010.04.015 20920888

[B28] Nakajima S. Inoue Y. Sasai T. Okajima I. Komada Y. Nomura T. (2014). Impact of frequency of nightmares comorbid with insomnia on depression in Japanese rural community residents: a cross-sectional study. *Sleep Med.* 15 371–374. 10.1016/j.sleep.2013.11.785 24560189

[B29] Nielsen T. Paquette T. Solomonova E. Lara-Carrasco J. Colombo R. Lanfranchi P. (2010). Changes in cardiac variability after REM sleep deprivation in recurrent nightmares. *Sleep* 33 113–122. 10.1093/sleep/33.1.113 20120628 PMC2802238

[B30] Rek S. Sheaves B. Freeman D. (2017). Nightmares in the general population: identifying potential causal factors. *Soc. Psychiatry Psychiatr. Epidemiol.* 52 1123–1133. 10.1007/s00127-017-1408-7 28712041 PMC5581821

[B31] Reynolds C. F. III Kupfer D. J. Hoch C. C. Stack J. A. Houck P. R. Berman S. R. (1986). Sleep deprivation in healthy elderly men and women: effects on mood and on sleep during recovery. *Sleep* 9 492–501. 10.1093/sleep/9.4.492 3809863

[B32] Rupp T. L. Wesensten N. J. Bliese P. D. Balkin T. J. (2009). Banking sleep: realization of benefits during subsequent sleep restriction and recovery. *Sleep* 32 311–321. 10.1093/sleep/32.3.311 19294951 PMC2647785

[B33] Sandman N. Valli K. Kronholm E. Revonsuo A. Laatikainen T. Paunio T. (2015). Nightmares: risk factors among the Finnish general adult population. *Sleep* 38 507–514. 10.5665/sleep.4560 25325474 PMC4355890

[B34] Sateia M. J. (2014). International classification of sleep disorders-third edition. *Chest* 146 1387–1394. 10.1378/chest.14-0970 25367475

[B35] Scarpelli S. D’Atri A. Bartolacci C. Gorgoni M. Mangiaruga A. Ferrara M. (2020). Dream recall upon awakening from non-rapid eye movement sleep in older adults: electrophysiological pattern and qualitative features. *Brain Sci.* 10:343. 10.3390/brainsci10060343 32503215 PMC7349242

[B36] Scarpelli S. D’Atri A. Mangiaruga A. Marzano C. Gorgoni M. Schiappa C. (2017). Predicting dream recall: EEG activation during NREM sleep or shared mechanisms with wakefulness? *Brain Topogr.* 30 629–638. 10.1007/s10548-017-0563-1 28434101

[B37] Schredl M. (2010). Nightmare frequency and nightmare topics in a representative German sample. *Eur. Arch. Psychiatry Clin. Neurosci.* 260 565–570. 10.1007/s00406-010-0112-3 20229263

[B38] Spielman A. J. Saskin P. Thorpy M. J. (1987). Treatment of chronic insomnia by restriction of time in bed. *Sleep* 10 45–56. 3563247

[B39] Spoormaker V. I. Montgomery P. (2008). Disturbed sleep in post-traumatic stress disorder: secondary symptom or core feature? *Sleep Med. Rev.* 12 169–184. 10.1016/j.smrv.2007.08.008 18424196

[B40] Tempesta D. Curcio G. De Gennaro L. Ferrara M. (2013). Long-term impact of earthquakes on sleep quality. *PLoS One* 8:e55936. 10.1371/journal.pone.0055936 23418478 PMC3572187

[B41] Tu Z. H. He J. W. Zhou N. (2020). Sleep quality and mood symptoms in conscripted frontline nurse in Wuhan, China during COVID-19 outbreak: a cross-sectional study. *Med. (Baltimore)* 99:e20769. 10.1097/md.0000000000020769 32590755 PMC7328950

[B42] Usami M. Iwadare Y. Kodaira M. Watanabe K. Aoki M. Katsumi C. (2013). Sleep duration among children 8 months after the 2011 Japan earthquake and tsunami. *PLoS One* 8:e65398. 10.1371/journal.pone.0065398 23738015 PMC3667796

[B43] van Liempt S. van Zuiden M. Westenberg H. Super A. Vermetten E. (2013). Impact of impaired sleep on the development of PTSD symptoms in combat veterans: a prospective longitudinal cohort study. *Depress. Anxiety* 30 469–474. 10.1002/da.22054 23389990

[B44] van Wyk M. Solms M. Lipinska G. (2019). Increased awakenings from non-rapid eye movement sleep explain differences in dream recall frequency in healthy individuals. *Front. Hum. Neurosci.* 13:370. 10.3389/fnhum.2019.00370 31680920 PMC6803546

[B45] Williamson J. B. Heilman K. M. Porges E. C. Lamb D. G. Porges S. W. (2013). A possible mechanism for PTSD symptoms in patients with traumatic brain injury: central autonomic network disruption. *Front. Neuroeng.* 6:13. 10.3389/fneng.2013.00013 24391583 PMC3867662

[B46] Zadra A. Donderi D. C. (2000). Nightmares and bad dreams: their prevalence and relationship to well-being. *J. Abnorm. Psychol.* 109 273–281. 10.1037/0021-843x.109.2.273 10895565

[B47] Zhang Y. Ren R. Sanford L. D. Yang L. Zhou J. Zhang J. (2019). Sleep in posttraumatic stress disorder: a systematic review and meta-analysis of polysomnographic findings. *Sleep Med. Rev.* 48:101210. 10.1016/j.smrv.2019.08.004 31518950

